# Editorial: Reading acquisition of Chinese as a second/foreign language

**DOI:** 10.3389/fpsyg.2023.1185195

**Published:** 2023-06-23

**Authors:** Linjun Zhang, Zaizhu Han, Yang Zhang

**Affiliations:** ^1^School of Chinese as a Second Language, Peking University, Beijing, China; ^2^State Key Laboratory of Cognitive Neuroscience and Learning, Beijing Normal University, Beijing, China; ^3^Department of Speech-Language-Hearing Sciences and Masonic Institute for the Developing Brain, University of Minnesota, Minneapolis, MN, United States

**Keywords:** reading acquisition of Chinese, second/foreign language, Chinese characters, orthographic knowledge, handwriting, morphological awareness

This editorial draws attention to the distinctive properties of the Chinese writing system and the difficulties it presents for individuals learning Chinese as a second/foreign language (CSL/CFL). Following the brief introduction, a summary overview is provided for the 19 submitted articles for this Research Topic, highlighting the contributions of these articles toward a better understanding of the universal and language-specific mechanisms in acquiring reading skills in a second language (Koda, 2007; Verhoeven and Perfetti, 2017).

The Chinese logographic writing system^1^ (Sproat and Gutkin, 2021) has a number of distinct properties that set it apart from alphabetic orthographies. First, each Chinese character occupies a square-shaped space (e.g., 学, which means “to study”) in sharp contrast to the linear structure of an alphabetic word. Chinese characters are composed of basic strokes (e.g., 一) and complex stroke patterns (e.g., 乙), which are completely different from graphemes of other languages in appearance. In fact, Chinese script has been found to have the greatest visual complexity among 131 writing systems (Chang et al., 2016, 2018). Second, each Chinese character corresponds to a syllable, rather than phoneme(s), which is quite distinct from grapheme-to-phoneme correspondences that are universal in alphabetic systems (McBride and Wang, 2015). Chinese strokes are not phonemic representations and are unpronounceable in contrast to Korean script, whose characters represent syllables with visual resemblance of Chinese script but the symbols within each Korean character represent phonemes (Li et al., 2022). Moreover, the phonetic component of a compound Chinese character provides unreliable information about pronunciation. Therefore, phonological information of a Chinese character is obtained via direct access to its phonological representation stored in the lexicon rather than by assembling phonemes. Third, there are a great number of homographic morphemes in Chinese because of the correspondence between the character (the basic orthographic unit) and the morpheme (the basic semantic unit). For example, the character “草” represents several morphemes, including “grass”, “haste” and “draft”. Most Chinese words are compound words composed of two or three characters with the exact meaning of each character (i.e., morpheme) disambiguated in the word context (e.g., which morpheme “草” represents is clear in “草原”, “草率” and “草稿”). Fourth, unlike alphabetic writing systems with spaces to clearly mark word boundaries, Chinese text does not use inter-character or inter-word spaces. Specifically, Chinese does not use space or any other visual marks to signify word boundaries and characters are presented contiguously regardless of whether two or more characters form a word or they belong to different words.

Researchers have long examined whether and how Chinese script-specific properties require specific perceptual and cognitive mechanisms for the development of efficient reading (Zhou and Marslen-Wilson, 1999; Zhou et al., 2009). Studies on adult and child native Chinese speakers have consistently shown that visual-orthographic knowledge and morphological awareness play important roles in Chinese reading due to the structural complexity and the existence of a large number of homographic morphemes (Shu et al., 2006; Zhang et al., 2023). Despite the lack of correspondence between strokes and phonemes, phonological awareness, which is the core linguistic subskill underlying alphabetic reading, also contributes to Chinese reading development (Ruan et al., 2018).

The distinctive characteristics of the Chinese writing system make learning to read Chinese a significant challenge for CSL/CFL learners. However, compared with the substantial amount of research on Chinese reading acquisition in native Chinese speakers, only a limited number of studies have investigated the acquisition of L2 Chinese reading skills. It remains unclear how CSL/CFL learners from different linguistic backgrounds acquire the knowledge of Chinese orthography (e.g., the intricate strokes and square configurations), syllable-character correspondence, morphological awareness and word segmentation without inter-word spacing. Moreover, there is a lack of empirical studies on how developing these linguistic skills affects CSL/CFL learners' ability to read sentences and passages at different Chinese proficiency levels. This Research Topic comprises 18 original studies and a systematic review that examined CSL/CFL learners' reading acquisition at various levels (i.e., character, word, sentence, and passage) from different L1 backgrounds.

Three papers focused on Chinese character writing due to the close relationship between reading and writing of characters for the logographic characteristics of Chinese script (Ziegler, 2006). One study conducted by Zhang investigated the structure of orthographic representations during character writing and found that various representational levels (character, logographeme, and stroke) were active simultaneously but logographeme was dominant. Chai and Ma used big data from 74362 CSL/CFL learners with 67 L1 backgrounds who took the HSK (Chinese Proficiency Test) to explore the relationship between character writing and sentence/passage reading. They discovered that character writing helped to overcome negative transfer from learners' L1s and interactively contributed to reading development in relation to language distance. Another study by Lau et al. adopted a delayed character copying task to measure Chinese orthographic knowledge in Vietnamese CSL/CFL learners. They found that learners chunked characters into functional units when they wrote, and the use of large (radical boundary) and small (logographeme boundary) grain-size units was affected by character reading ability.

Two papers explored methods for improving the teaching and learning of Chinese characters by enhancing visual-orthographic processing skills. Hou and Jiang investigated the effectiveness of radical- and stroke-targeted teaching methods for native alphabetic language speakers but found that both methods had negative effects on character reading. These results suggest that analytic processing strategies might undermine holistic processing required for character reading. However, Chang et al. reported contradictory results by demonstrating that different methods of presenting characters, including the stroke-targeted method used by Hou and Jiang, had positive effects on character reading. As the two studies differed in terms of participants (native English speakers versus native speakers of various L1 backgrounds) and materials (simplified versus traditional characters), further research is needed to determine how the analytic teaching method can help CSL/CFL learners read and write Chinese characters.

To assess the reading ability of CSL/CFL learners, a commonly used method is the character recognition test, although studies differ in which specific measurement is used. Zhang, Kim et al. compared three typical character recognition measurements (i.e., phonological, semantic and phonological + semantic) and found that each measurement yielded different predictions for Chinese proficiency depending on the leaners' L1 backgrounds. This suggests that future studies need to consider the L1 backgrounds of CSL/CFL leaners when selecting which measurement to use.

Five studies investigated the role of linguistic subskills and general cognitive ability in L2 Chinese reading at word and sentence/passage levels. A meta-analysis by Chen and Zhao found a moderate relationship between phonological awareness and word reading, despite the logographic characteristics of Chinese script. Two studies by Chen et al. and Zhang, Zhang et al. respectively examined the contribution of morphological awareness at radical and character levels to sentence/passage reading comprehension, and confirmed the important role of grapho-morphological awareness in L2 Chinese reading acquisition. Zhou chose to investigate the role of syntactic awareness, particularly word order knowledge, which had been paid little attention to in previous studies, and found that it made a unique contribution to passage reading even when other reading-related subskills were controlled for. Xie et al. studied the contribution of general cognitive ability, and found that the cognitive control predicted sentence reading comprehension, suggesting that theoretic models of L2 Chinese reading need to include cognitive control skills as additional predictors.

Two papers studied the importance of word segmentation and inter-word space for L2 Chinese reading. Hao et al. showed that word segmentation and word-meaning access were crucial for reading accuracy in both high and low proficiency learners. Cui conducted an eye-tracking experiment and found that adding inter-word space improved reading efficiency of connected passages in beginning CSL/CFL learners. These findings suggest that providing inter-word space can serve as a useful pedagogical tool to improve sentence/passage reading by helping learners, particularly beginning learners segment and identify words.

Four papers addressed new areas in L2 Chinese acquisition that have been seldom studied. Wu et al. examined the influence of L1 transfer on complex syntactic representations. Tamaoka and Zhang studied the acquisition of temporal adverbs by native Japanese speakers and found that L2 Chinese proficiency affected the placement of these words. Lu et al. focused on splitable compound words and reported that split presentation of the words significantly hindered the performance of native Spanish speakers. Lastly, Wang et al. investigated the effect of positive valence bias on the acquisition of Chinese emotion idioms and discovered that it had an impact on the initial learning phase.

In addition to behavioral research, two studies adopted neuroimaging techniques to investigate semantic processing during L2 Chinese reading. Li et al. used electroencephalography (EEG) recordings to explore how Chinese-Malay bilingual speakers with Chinese as their heritage language integrated meaning in Chinese classifier-noun phrases. They found similarities and differences between the bilinguals and monolingual Chinese speakers, indicating that bilinguals differ to some extent in semantic prediction and integration during the processing of classifier-noun agreement. Lai et al. used functional magnetic resonance imaging (fMRI) to study the neural mechanisms underlying semantic judgment of Chinese characters in high-proficient CSL/CFL learners. The fMRI results revealed less activations in temporal regions but greater activations in occipital regions in the L2 group relative to the L1 control group, indicating that the CSL/CFL learners relied more on orthographic processing when their ability to access semantic information was limited.

Collectively, these studies address key issues in L2 Chinese reading acquisition and provide new insights into orthographic, phonological, and semantic processing of Chinese characters as well as the contributions of these linguistic subskills and cognitive skills to sentence/passage reading. These studies adopted various behavioral and neuroimaging (EEG and fMRI) methods with appropriate experimental design. They collectively constitute a valuable sample of current research on L2 Chinese reading acquisition, demonstrating the importance of integration of various research methods for future investigations. Given that theories of L2 reading acquisition are primarily based on alphabetic scripts, current and future studies in Chinese script play important roles in understanding the universal and language-specific mechanisms for L2 reading acquisition. This Topic provides valuable insights for readers who are interested in this field.

## Author contributions

LZ and ZH drafted the editorial. YZ revised the editorial. All authors contributed to the article and approved the submitted version.

## Footnotes

^1^Both “logographic” and “morphosyllabic” are used in the literature to describe the properties of the Chinese writing system. Specifically, “logographic” is used to emphasize that identically pronounced but semantically contrastive elements have distinct graphic representations and “morphosyllabic” is used to emphasize that each Chinese character denotes a syllable as well as some aspect of the morpheme. The two terms are not mutually exclusive and the Chinese script can be described as both logographic and morphosyllabic in taxonomies. We choose the term “logographic” to highlight the uniqueness of Chinese scripts, i.e., the majority of Chinese characters carry constituent graphemes that are logographic. That's not a characteristic shared by alphabetic (e.g., English) or syllabic (e.g., Korean) writing systems. The term “morphosyllabic” does not carry the same distinctive appeal.

## References

[B1] Chang L. Y. Chen Y. C. Perfetti C. A. (2018). GraphCom: a multidimensional measure of graphic complexity applied to 131 written languages. Behav. Res. Methods. 50, 427–449. 10.3758/s13428-017-0881-y 28425059

[B2] Chang L. Y. Plaut D. C. Perfetti C. A. (2016). Visual complexity in orthographic learning: modeling learning across writing system variations. Sci. Stud. Read. 20, 64–85. 10.1080/10888438.2015.1104688

[B3] Koda K. (2007). Reading and language learning: crosslinguistic constraints on second language reading development. Lang. Learn. 57, 1–44. 10.1111/0023-8333.101997010-i1

[B4] Li X. Huang L. Yao P. Hyönä J. (2022). Universal and specific reading mechanisms across different writing systems. Nat. Rev. Psychol. 1, 133–144. 10.1038/s44159-022-00022-6

[B5] McBride C. Wang Y. (2015). Learning to read Chinese: universal and unique cognitive cores. Child Dev. Perspect. 9, 196–200. 10.1111/cdep.12132

[B6] Ruan Y. Georgiou G. K. Song S. Li Y. Shu H. (2018). Does writing system influence the associations between phonological awareness, morphological awareness, and reading? A meta-analysis. J. Educ. Psychol. 110, 180–202. 10.1037/edu0000216

[B7] Shu H. McBride-Chang C. Wu S. Liu H. Y. (2006). Understanding Chinese developmental dyslexia: morphological awareness as a core cognitive construct. J. Educ. Psychol. 98, 122–133. 10.1037/0022-0663.98.1.122

[B8] Sproat R. Gutkin A. (2021). The taxonomy of writing systems: how to measure how logographic a system is. Comput. Linguist. 47, 477–528. 10.1162/COLI_a_0040935410909

[B9] Verhoeven L. Perfetti C. A. (2017). Learning to Read Across Languages and Writing Systems. Cambridge: Cambridge University Press.

[B10] Zhang L. Xia Z. Zhao Y. Shu H. Zhang Y. (2023). Recent advances in Chinese developmental dyslexia. Annu. Rev. Linguist. 9, 439–461. 10.1146/annurev-linguistics-030421-065648

[B11] Zhou X. Marslen-Wilson W. (1999). Phonology, orthography, and semantic activation in reading Chinese. J. Mem. Lang. 41, 579–606. 10.1006/jmla.1999.2663

[B12] Zhou X. Ye Z. Cheung H. Chen H. (2009). Processing the Chinese language: an introduction. Lang. Cogn. Neurosci. 24, 929–946. 10.1080/01690960903201281

[B13] Ziegler J. C. (2006). Do differences in brain activation challenge universal theories of dyslexia? Brain Lang. 98, 341–343. 10.1016/j.bandl.2005.05.002 16040110

